# Bipolar disorder and dopamine dysfunction: an indirect approach focusing on tardive movement syndromes in a naturalistic setting

**DOI:** 10.1186/1471-244X-9-16

**Published:** 2009-04-28

**Authors:** Inge van Rossum, Diederik Tenback, Jim van Os

**Affiliations:** 1 Eli Lilly Nederland, Medical Department, Houten, the Netherlands; 2 Symfora Group Psychiatric Center, Utrechtseweg 266, 3818 EW Amersfoort, the Netherlands; 3 Department of Psychiatry, University Medical Center Utrecht, Heidelberglaan 100, 3584 GX Utrecht, the Netherlands; 4 Department of Psychiatry and Neuropsychology, Maastricht University, Maastricht, the Netherlands; 5 Division of Psychological Medicine, Institute of Psychiatry, London SE5 8AF, UK

## Abstract

**Background:**

It has been suggested that dopamine dysfunction may play a role in bipolar disorder (BD). An indirect approach to examine this issue was developed, focusing on associations between dopamine proxy measures observed in BD (dopamine-related clinical traits using tardive movement syndromes as dopamine proxy measure of reference).

**Methods:**

3459 eligible bipolar patients were enrolled in an observational study. Incidence rates of tardive movement syndromes (tardive dyskinesia and tardive dystonia; TDD) were examined. A priori hypothesized associations between incident TDD and other dopamine proxies (e.g. prolactin-related adverse effects, bipolar symptoms) were tested over a 2 year follow-up period.

**Results:**

The incidence rate of tardive syndromes was 4.1 %. Incident TDD was independently associated not only with use of antipsychotics, but also with more severe bipolar symptoms, other extrapyramidal symptoms and prolactin-related adverse effects of medication.

**Conclusion:**

Apart from the well-known association with antipsychotics, development of TDD was associated with various other dopamine proxy measures, indirectly supporting the notion of generalised dopamine dysregulation in BD.

## Background

Extrapyramidal symptoms (EPS) in general and tardive dyskinesia (TD) in particular have been extensively studied in schizophrenia. Even though a number of studies suggest that bipolar patients experience higher rates of EPS (parkinsonism, dystonia, akathisia) and TD compared to patients with a diagnosis of schizophrenia [1,2], research within the bipolar disorder (BD) population has been limited.

Berk and colleagues, reviewing converging data on the role of dopamine (DA) in bipolar disorder, recently postulated the Dopamine Dysregulation Syndrome [3], based on a consilience approach using research from a diversity of domains, including preclinical, genetic, neuroimaging, pharmacological and clinical research. The model proposes a cyclical dysregulation in quantitative DA transmission in BD, the manic phase being associated with an increase in DA transmission. Secondary down regulation of key elements and pre- as well as post-synaptic receptors may subsequently decrease DA transmission, dampening the cycle. A substantial reduction in DA transmission is hypothesized to characterize the depressive phase, which may be alleviated by secondary up-regulation of the same key elements. Up and down regulation may be mediated by endogenous homeostatic mechanisms; individual vulnerabilities exist to the hyper- and hypodopaminergic state, explaining the large inter-individual variability in illness course [3].

While of major interest, hypotheses about alterations in DA function in severe mental illness are difficult to examine directly. It has been suggested, therefore, that alterations in DA function in severe mental illness (i.e. illness liabilities affecting various aspects of dopamine signaling, separate from effects occasioned by antipsychotic medications) may be explored using clinical variables serving as proximity measures (i.e. indirect measures) for DA neurotransmission in different DA tracts. These may include, for example, presence of (i) prolactin-related adverse effects as a proxy for alterations in the tuberoinfundibular tract [4], ii) TD and EPS as proxies for alterations in the nigrostriatal DA tract [5] whereas (iii) psychotic [5,6], manic [7] and depressive [8] symptoms may reflect altered mesolimbic DA neurotransmission. Using these proxy measures in a prospective study of schizophrenia patients, Tenback and colleagues showed associations between incidence of TD on the one hand and occurrence of EPS [9], prolactin-related adverse effects [4] and worsening of psychotic symptoms on the other [6], suggesting general DA dysregulation across the different DA tracts in schizophrenia.

The Dopamine Dysregulation Syndrome, as applied to BD by Berk and colleagues [3], is currently limited to DA functioning in the mesolimbic tract, focusing on mania and depression. Similar to schizophrenia, associations between the proxies representing alterations in the different DA tracts may be hypothesized for bipolar disorder as well. In order to test this hypothesis, DA dysfunction was examined in a sample of patients with bipolar disorder, using the proxy measures defined by Tenback et al [4,6]. In line with Tenback and colleagues [4,6], tardive movement syndromes formed the reference outcome for the analyses. Incidence rates of tardive extrapyramidal syndromes were calculated, and associations between these syndromes and proxy measures for alterations in other DA tracts were examined.

## Methods

### Study design and population

EMBLEM (European Mania in Bipolar Evaluation of Medication) was a 2-year prospective, observational study on the outcome of pharmacological treatment of mania across 14 European countries. A total of 3459 eligible in- and outpatients were enrolled at the discretion of the treating psychiatrist. Patients were eligible for participation if they were at least 18 years old and they initiated/changed oral medication for treatment of acute mania in bipolar disorder (antipsychotics, anticonvulsants and/or lithium; not antidepressants or benzodiazepines) within the standard course of care. During the acute treatment phase, assessments took place at baseline and 1, 2, 3, 6 and 12 weeks after baseline. The maintenance phase consisted of assessments at 6, 12, 18, and 24 months after baseline. ERB approval and patient informed consent were obtained according to local legal requirements. The study design has been described in detail in previous reports [10,11].

### Extrapyramidal symptoms

Investigators were requested to assess presence and severity of parkinsonism, akathisia, dystonia and TD that they judged to be associated with medications used to treat bipolar disorder. This assessment was based on the investigator's clinical experience and judgment and rated as follows: 0 = not present; 1 = present, but does not significantly interfere with patient's functioning; 2 = present, and significantly interferes with patient's functioning. Guided by previous analyses using these measures [4], movement disorder variables were analyzed as dichotomous indicators (0 = not present versus 1 = present; the latter combining scores of '1' and '2'). These assessments were performed at baseline and all subsequent visits.

The scales used to measure extrapyramidal symptoms were simple and did not include instructions on or specific anchors for differential diagnosis. Therefore, in order to avoid diagnostic misclassification, only persistent dystonia and TD were used in the current analyses. Using the persistence measure ensured differentiation from the acute syndromes, as, for example, acute dystonia would be unlikely to persist over two subsequent visits whereas tardive dystonia would. Persistence was defined as the presence of the individual syndromes over at least 2 consecutive visits. Thus, the variable "persistent dystonia" was rated as follows: 0 = no or acute/incidental dystonia; 1 = persistent dystonia. Similarly, "persistent TD" was rated as 0 = no or incidental TD; 1 = persistent TD. Persistent TD and persistent dystonia were analyzed together as a single group (hereafter TDD: 0 = no persistent TD or persistent dystonia present, 1 = persistent TD and/or persistent dystonia present). The rational for combining TD and dystonia comes from (i) their strong association [12,13], (ii) shared risk factors and mechanisms [14,15] and (iii) the fact that existing scales measuring tardive syndromes do not differentiate between TD and tardive dystonia [16,17]. Parkinsonism and akathisia were also compiled into a single variable, hereafter named EPS (0 = neither parkinsonism nor akathisia present, 1 = parkinsonism and/or akathisia present).

### Incidence of TDD

Incidence rates of TDD were determined by allocating each patient person-time for the TDD outcome according to the interval from baseline to the visit in which a patient was diagnosed with TDD. If no such diagnosis was made, the interval covered baseline to the final visit of each patient. Nine time bands were constructed (baseline – week 1; week 1 – week 2; week 2 – week 3; week 3 – 6 weeks; 6 weeks – 3 months; 3 months – 6 months; 6 months – 12 months; 12 months – 18 months; 18 months – 24 months). The incidence of TDD was calculated by dividing the total number of incident cases of TDD by the total person-years. The same procedures were followed for calculating separate incidence rates for tardive dystonia and TD. All analyses were conducted in the risk set of patients free of TDD at baseline.

### Associations between clinical factors and incident TDD

Cox proportional hazard regression was used to assess survival time without TDD associated with various time-varying clinical variables. The following clinical measures were used as proxies for DA dysregulation:

(i) Psychotic and manic symptoms may be associated with high DA transmission in the mesolimbic pathway [5,7]. Depression may be associated with lower DA transmission in the same tract, even though different receptor classes or sub-regions may be involved [5,8]. In the current analyses, the CGI-BP severity of mania, CGI Hallucinations/delusions and CGI-BP depression were regarded as proxy measures for altered DA transmission within the mesolimbic DA tract. In addition, the CGI-BP overall illness was used as an overall measure of dysregulation in this tract. All CGI scores were rated for severity on a seven-point scale [18] and used at each visit including baseline.

(ii) Both amenorrhea and sexual dysfunction are associated with elevated prolactin levels induced by low DA transmission [19,20] originating in the tuberoinfundibular DA tract. This link is likely stronger for amenorrhea, as sexual disturbances in patients with schizophrenia are of multifactorial origin, and are therefore only in part attributable to illness- or medication-related prolactin levels [19,20]. Presence of amenorrhea and sexual dysfunction were used as proxy measures for altered DA transmission in the tuberoinfundibular tract (both defined as 0 = not present; 1 = present; measured at each visit).

(iii) Extrapyramidal symptoms, including TD, have been hypothesized to reflect low DA transmission in the nigrostriatal DA tract in the brain [5]. Research indicates that EPS (defined as parkinsonism, akathisia and acute dystonia) represents a vulnerability to develop tardive movement disorders, in particular tardive dyskinesia, in patients with schizophrenia [9].

Therefore, presence of EPS as a proxy measure for dysfunctional DA transmission in the nigrostriatal tract, was tested for association with incident TDD.

(iv) Use of antipsychotics (APs) is known to affect dopamine transmission [21] and in addition is strongly associated with TD [5]. Use of AP was assessed at each visit, and included in the analyses (0 = no AP use, 1 = first generation antipsychotic (FGA), 2 = second generation antipsychotic (SGA)).

The four clusters of proxy measures for DA dysfunction (bipolar symptoms, prolactin-related adverse effects, EPS and use of antipsychotics) were individually included as independent variables in the Cox models in order to determine associations with incident TDD. Finally, all variables were entered simultaneously in the model in order to determine which associations persisted independently of other factors. Effect sizes were expressed as Hazard Ratio's (HR) and 95% confidence intervals. The two-sided significance level was 5%.

### Adjustment by propensity score

Analyses were performed with and without confounders (adjusted and unadjusted analyses, respectively). For each analysis, all patients with non-missing values on the dependent and independent variables were included, as well as on all confounding variables in case of adjusted analyses. Confounders were based on a review of the literature within patient populations diagnosed with bipolar disorder, schizophrenia or psychotic disorders in general. The following confounders were introduced in the Cox regression models: social economic status (SES, expressed as educational achievement; 1 = no education, 2 = primary school, 3 = secondary school lower, 4 = secondary school upper, 5 = post-secondary vocational training, 6 = university), country, compliance (0 = no medication prescribed or always complies; 1 = never complies or 50% of the time), age per decade [22,23], age of onset in years [24], gender [23,25] and duration of illness in years [26]. As a decrease in TDD incidence over time was anticipated, analyses were also adjusted for visit number.

It is common practice to increase the dosages of antipsychotics or lithium in response to increased symptom severity. It is widely accepted that antipsychotic use and lithium in itself are associated with an increased risk for developing movement disorders and other adverse effects [5]. Thus, associations between higher symptom severity or the presence of adverse effects with a higher incidence of TDD may represent a confounding effect of AP or lithium use or dose burden. Therefore, except when testing associations between TDD and the use of antipsychotics, multiple treatment-related variables were included as confounders in order to eliminate spurious results for dopamine abnormalities related to the (changes in) use of antipsychotics and lithium. The following treatment-related time-varying variables were included as confounders: (i) use of APs (dichotomous variable: 0 = no use of AP, 1 = use of FGA and/or SGA); (ii) dichotomous variables indicating use (0 = no use, 1 = use) of the following individual treatments; amisulpride, clozapine, haloperidol, olanzapine, quetiapine, risperidone, ziprazidone, other AP or lithium (iii) dose of treatment used, expressed as dose equivalents; (iv) change in dose of treatment with respect to the previous visit, expressed as dose equivalents. In addition, except when testing for an association between the CGI-BP Overall illness and incident TDD, *change *in CGI-BP Overall illness score relative to the previous visit was included as confounder.

As many confounding variables were included in the models, traditional control for confounding by inclusion of covariates in the model may not be sufficient, as the degree of 'control' afforded by such models depends on the overlap in characteristics between the two outcome groups. The use of the propensity score has been suggested as a means to obtain more complete control in these circumstances [27]. The propensity score for an individual, defined as the conditional probability of (in this case) developing TDD given the individual's covariates, can be used to balance the covariates in observational studies, and thus reduce bias [28]. In other words, by using propensity scores, a collection of covariates is replaced by a single covariate, being a function of the original ones, while minimizing the loss of degrees of freedom. As the propensity score model could not create sufficient balance between the groups due to the variable 'haloperidol', haloperidol was not included in the propensity score model and adjusted for separately in the model, together with the dependent variable, the independent variable under investigation and the propensity score representing the other specified confounders.

Not all countries participated in the maintenance phase of the study (12 weeks onwards), resulting in a decrease in sample size after the 12 weeks (Switzerland, Denmark, Germany and Spain only participated in the acute phase). Apart from the decrease in overall sample size, the samples for the individual analyses varied somewhat on the basis of the availability of complete data for variables included in the separate models. All analyses were performed using the computer package STATA, version 10.0 [29].

### TDD validity: sensitivity analyses

Additionally, sensitivity analyses were conducted using a stricter criterion for incidence in order to exclude any possibility of bias due to carry-over from influences occasioned by factors acting during the period before baseline. To this end, a stricter risk set was defined as the sample of patients free from dystonia or TD at baseline *as well as *at visit 2 (one week post-baseline). First occurrence of any incident tardive syndrome could therefore occur at visit 3 (two weeks post-baseline), while for the purpose of the current analyses incidence could first occur at visit 4, due to the requirement of persistence of symptoms for at least 2 consecutive visits. Consequently, misclassification of the acute form of dystonia, which usually has an onset within 5 days of new antipsychotic treatment [30], could be ruled out with even more confidence.

## Results

A total of 3459 subjects participated in the EMBLEM study and fulfilled the CGI-BP eligibility criteria (CGI-BP mania ≥ 3). 355 Patients were excluded from the analyses, either due to missing baseline ratings of dystonia and/or TD, or due to presence of dystonia and/or TD at baseline. Of the remaining 3104 patients, 43.5% were male and the mean age was 44.5 years (sd 13.4). The mean age of onset was 29.9 years (sd 11.1). Table 1 provides an overview of the baseline clinical characteristics of the patient sample. As a frame of reference, the medication used at enrollment, as well as the medication prescribed at baseline are presented in Table 2. More than half of the patients did not use any antipsychotic medication at enrollment, while 30% used antidepressants at that time. Second generation APs were prescribed most frequently at the baseline visit, followed by anticonvulsants.

**Table 1:** Baseline clinical characteristics of the total sample (n = 3104).

Inpatient status (%)	40.0%
Rapid cycling (4 ≤ episodes per year; %)	17.2%
CGI-BP overall illness (mean, sd)	4.7 (1.0)
CGI-BP mania (mean, sd)	4.8 (1.0)
CGI Hallucinations/delusions (mean, sd)	2.9 (1.8)
CGI-BP depression (mean, sd)	1.8 (1.2)

**Table 2:** Medications at presentation and prescribed on baseline visit for the complete sample (n = 3104).

	At presentation on baseline visit	Prescribed at baseline visit†
No AP (%)	53.2%	11.1%
FGA (%)	25.4%	11.5%
SGA (%)	15.4%	65.3%
Combination of FGA and SGA (%)	6.0%	12.1%
Lithium (%)	18.8%	25.1%
Anticonvulsants (%)	29.9%	49.1%
Antidepressants (%)	30.0%	15.6%
Anticholinergics (%)	*	8.8%

FGA = first generation antipsychotic; SGA = second generation antipsychotic; *not measured; †all patients were prescribed a new treatment at baseline; medication use is extensive in this population, therefore not all used medication is included in this table (e.g. benzodiazepines).

### Incidence rate

The TDD incidence was calculated by dividing the total number of incident cases of TDD by the total person-years. Over two years of treatment, the sample contributed 3163 patient years, while there were 129 new cases of TDD, yielding an incidence rate of 4.1% (95% CI: 3.4, 4.8). The separate incidence rates for TD and Tardive Dystonia were 1.0% (95% CI: 0.7, 1.4) and 3.3% (95% CI: 2.7, 4.0) respectively. Overall, incidence rates decreased with time, as illustrated by Table 3, where incidence rates are provided per time band.

**Table 3:** Incidence of tardive dystonia, tardive dyskinesia and TDD for each time band (n = 3104).

	Tardive dystonia			TD			TDD		
	N	Person-years	Incidence Rate	N	Person-years	Incidence Rate	N^a^	Person-years	Incidence Rate
Visit 3 (week 2)	37	114	32.6%	7	114	6.2%	41	114	36.1%
Visit 4 (week 3)	23	56	40,7%	4	57	7.0%	25	56	44.3%
Visit 5 (week 6)	15	141	10.6%	2	144	1.4%	17	141	12.0%
Visit 6 (month 3)	12	287	4.2%	4	293	1.4%	15	286	5.2%
Visit 7 (month 6)	5	440	1.1%	4	454	0.9%	8	439	1.8%
Visit 8 (month 12)	6	753	0.8%	5	776	0.6%	10	750	1.3%
Visit 9 (month 18)	2	721	0.3%	1	746	0.1%	3	716	0.4%
Visit 10 (month 24)	5	666	0.8%	6	688	0.9%	10	661	1.5%

The time band between visit 1 and 2 was not included, due to the definition of persistence (presence of symptoms for at least 2 consecutive visits). ^a^As some patients may present with both tardive dystonia and TD, N for TDD may be lower than the sum of N for tardive dystonia and TD.

### Associations between tardive dystonia and TD

In order to examine the validity of combining the tardive dystonia and TD variables into a combined outcome, associations between these tardive movement disorders were calculated using Cox analyses. The probability of incident tardive dystonia in the presence of TD was high (HR = 9.56, 95% CI = 6.02, 15.18; P < .001), while the probability of incident TD in the presence of tardive dystonia was also high (HR = 22.25, 95% CI = 9.20, 53.85; P < .001).

### Associations between clinical factors and incident TDD

Table 4 provides associations between incident TDD and hypothesized dichotomous variables; presence of sexual dysfunction, amenorrhea and EPS were found to be associated with incident TDD. Additionally, compared with no AP use, both FGAs and SGAs showed a stronger association with TDD. Associations between incident TDD and hypothesized bipolar symptom severity are presented in Table 5. Higher symptom severity was consistently found to be associated with incident TDD.

**Table 4:** Associations between incident TDD and various dichotomous clinical factors during a period of 2 years (n = 3104).

	TDD rate exposed			TDD rate non-exposed					
Clinical factor	N	Person years^a^	Rate (%)	N	Person years	Rate (%)	HR adjusted (95% CI)	HR unadjusted (95% CI)	Sensitivity analyses (HR adjusted) 95% CI)^b^
Sexual dysfunction	44	505	8.7	84	2637	3.2	2.68 (1.72, 4.16)*	2.72 (2.20, 3.37)*	2.61 (1.56, 4.39)*
Amenorrhea	11	126	8.7	107	2810	3.8	2.54 (2.10, 3.09)*	2.38 (1.74, 3.26)*	2.48 (1.83, 3.36)*
Extra-pyramidal symptoms	89	273	32.6	40	2875	1.4	13.94 (6.90, 28.19)*	17.20 (9.11, 32.47)*	17.79 (6.70, 47.26)*
FGA use vs no AP	31	287	10.8	9	768	1.2	2.64 (1.94, 3.60)*	2.64 (1.97, 3.53)*	2.32 (1.66, 3.24)*
SGA use vs no AP	69	1902	3.6	9	768	1.2	2.18 (1.20, 3.97)†	2.16 (1.02, 4.54)†	1.50 (0.79, 2.85)

*P ≤ 0.001; †P ≤ 0.05 N = min. 1143 (as amenorrhea analysis was limited to women), max. 2025 for the adjusted analyses; n = min. 1606, max. 2953 for the unadjusted analyses. ^a^Person years in follow-up. For instance, for sexual dysfunction, the patients included in the analyses contributed (505 + 2637=) 3142 years of follow-up time. 44 patients that developed TDD presented with comorbid sexual dysfunction (rate of 8.7%). 84 patients that developed TDD did not present with comorbid sexual dysfunction (rate of 3.2%). ^b^Sensitivity analyses were conducted with a stricter criterion for incidence TDD; a stricter risk set was defined as the sample of patients free from dystonia or TD at baseline *as well as *at visit 2 (one week post-baseline). Consequently, person-years included in this table do not hold for the sensitivity analyses.

**Table 5:** Associations between incident TDD and various continuous clinical factors over a 2-year period.

	HR adjusted (95% CI interval) *per CGI point*	HR unadjusted (95% CI interval) *per CGI point*	Sensitivity analysis HR adjusted (95% CI interval) *per CGI point*
CGI-BP Overall illness	1.59 (1.32, 1.90)*	1.49 (1.24, 1.79)*	1.69 (1.44, 1.79)*
CGI-BP Hallucinations/delusions	1.53 (1.37, 1.70)*	1.49 (1.35, 1.64)*	1.60 (1.42, 1.80)*
CGI Mania	1.56 (1.36, 1.78)*	1.47 (1.24, 1.73)*	1.69 (1.53, 1.87)*
CGI-BP Depression	1.38 (1.12, 1.71)*	1.30 (1.14, 1.49)*	1.45 (1.18, 1.78)*

*P ≤ .002. N = min. 2013, max. 2016 for the adjusted analyses; n = min. 2948, max. 2952 for the unadjusted analyses.

### Independence of associations with incident TDD

In order to test the degree of independence of the different associations between TDD and the various DA-proxies, a model with all predictors included together was examined. This revealed that the CGI-BP Hallucinations/delusions (HR *per CGI point *= 1.13, 95% CI: 1.01, 1.28; P = .041), sexual dysfunction (HR = 1.47, 95% CI: 1.12, 1.93; P = .006) and presence of EPS (HR = 13.33, 95% CI: 5.01, 35.50; P < .001) were associated with incident TDD independent of other factors.

### Sensitivity analyses

The sample for the sensitivity analyses consisted of 2657 patients. The incidence rate for dystonia decreased to 2.2%, TD to 0.8% and TDD to 2.9%. All but one association between DA-proxies and TDD remained significant and effect sizes did not change substantially; only the association between SGA use (versus no AP use) and TDD was slightly reduced and no longer significant. Results are included in Tables 4 &5.

## Discussion

One in twenty-five participants experienced new onset tardive dystonia and/or TD over a period of 2 years. Although carry-over effects from previous medications cannot be excluded because (i) the visits used to define persistence were close to each other in time and (ii) there was a linear decrease in incidence of TDD over the entire study period, the results from the sensitivity analyses suggest that carry-over effects did not play an important role. Indeed, even if carry-over effects existed, there is no reason to assume that these should per se affect the reported associations with third variables. Reported incidence rates of tardive syndromes in bipolar populations vary widely in the literature, probably due to variation in use of medication (type of antipsychotic, duration of exposure to lithium, polypharmacy), tardive syndrome definitions, characteristics of patient populations, study designs and mood state dependent fluctuations [2]. Indeed, the sensitivity analyses carried out for the purpose of the current analyses clearly show that subtle changes in definition affect incidence rates.

To our knowledge, rates of tardive dystonia have not been reported in a similar population. One of the few reports available on tardive dystonia originates from a sample of chronic psychiatric patients from Curacao, mostly suffering from schizophrenia. The reported incidences for tardive dystonia and TD were 0.7%, and 10.2%, respectively [13], whereas in our sample tardive dystonia had a higher incidence than TD. However, the Curacao study also reported that the incidence of tardive dystonia diminished over time, whereas the risk of TD followed an inverse pattern [30]. This may explain the discrepancy with our current findings, as the mean age in the current sample was lower (mean age 44 years, sd 13) compared to the chronically ill inpatient population from the Curacao study (mean age 53 years, sd 17). Van Harten and colleagues demonstrated that the rate of tardive dystonia was highest in the age group of 44 years or younger, whereas the rate of TD increased substantially after that age. Indeed, the incidence rate for tardive dystonia in the current study (3.3%; 2.3% when a stricter definition was applied), approximates the 3% incidence rate reported for patients on long-term antipsychotic treatment in a review by Van Harten et al. [31]. More research is needed within the bipolar spectrum to confirm the absolute risk of tardive syndromes in this specific population.

### Expanding the Dopamine Dysfunction Syndrome model?

A strong association was demonstrated between TDD incidence and various clinical factors, beyond antipsychotic medication, that were regarded as proxy measures for DA dysfunction in different tracts: high symptom severity scores, presence of sexual dysfunction, amenorrhea and EPS. Thus, (proxy) dysfunction in the nigrostriatal tract was found to be associated with (proxy) dysfunctions in the mesolimbic and the tuberoinfundibular DA tracts, indirectly suggesting generalised dopaminergic dysfunction.

The association between EPS and TDD was anticipated based on similar findings in schizophrenia [9], and the fact that extrapyramidal symptom clusters in general have been linked to low DA transmission in the nigrostriatal tract [5]. Associations between measures of sexual dysfunction and TDD are somewhat more complex as they imply the involvement of different tracts; they are, however, both associated with relatively low DA transmission [5,19,20].

Explaining the associations between more severe mania and psychotic symptoms on the one hand, and incident TDD on the other, is more complicated, as these symptom clusters seemingly represent high rather than low states of DA transmission, the latter being associated with TDD. To the best of our knowledge, an association between psychosis severity and TDD has not been reported before within a bipolar population. One might argue that the increase in psychotic symptoms and the emergence of TDD may be occasioned simultaneously by withdrawal of APs [32]. Alternatively, it may reflect increased dose of antipsychotics prescribed in response to increases in psychotic or manic symptoms, causing TDD. Although either of these confounding mechanisms cannot be completely excluded, it may be considered unlikely given that the current analyses were adjusted for changes in AP use and dose, as well as changes in CGI overall symptoms severity.

The association between more severe mania and TDD incidence may be considered surprising, as the limited reports available suggest a decrease of TD severity during manic episodes [2]. It may be attractive to speculate, in combination with the association found between TDD and psychotic symptoms, that this finding can be explained using the concept of 'supersensitivity psychosis', which postulates that psychotic symptoms may be produced by increased sensitivity of DA receptors in the mesolimbic tract [33]. Manic symptoms, similar to psychotic symptoms, have been linked to the mesolimbic DA tract [5,7], suggesting that this theory may be extrapolated to the mania symptom cluster as well. Although speculative, the hypothesis that the concept of supersensitivity may additionally extend to the nigrostriatal tract, could explain the associations found in the current study.

This hypothesis could also explain the finding of decreasing TDD incidence over time, as better BD symptom control might represent a dampening of supersensitivity, which might be extended to multiple tracts. Another explanation for this finding could be that patients who are highly sensitive to TDD development would develop the syndrome early in the study, whereas less sensitive patients either develop the syndrome at a later stage or not at all.

Even though the apparent contrast in the direction of proxy DA transmission status in the mesolimbic (up regulated) and nigrostriatal (down regulated) pathways that were associated with TDD is difficult to explain, it is compatible with the notion of broad dysfunction in the DA system. Alternatively, rather than an absolute interpretation of "high" or "low" states, DA instability or alterations in regulatory influences among the different DA tracts may instead represent the core characteristic driving the observed associations. The limited conclusion that can be drawn at this time is that more severe BD related symptoms are associated with an increased probability of tardive syndromes, the reason for which remains speculative.

### Limitations

The findings of the current study are subject to a number of limitations. First, we depended on proxy measures of DA transmission. The literature indicates that the investigated symptoms and adverse effects are, to a certain extent, related to DA dysfunction in certain areas. However, various external factors may influence these symptoms and effects without (necessarily) altering DA transmission. For instance, use of concomitant medication or antagonism of other receptors by APs may affect sexual dysfunction [19,20].

Second, if a pan-dopaminergic dysregulation exists within BD, it remains unknown what may cause such a state. For example, a reduction in sensitivity of postsynaptic DA receptors, altered pre-synaptic activity, a reduction in absolute or relative DA concentrations or another mechanism yet to be revealed may be involved. Obviously, epidemiological research is not the appropriate methodology to investigate these underlying mechanisms; more neuroimaging and pre-clinical data are needed to shed light on the nature and extent of DA dysfunction within the framework of the hypothesized dopamine dysregulation syndrome proposed by Berk and colleagues [3] as well as the extensions proposed in the current report.

Third, as cogently discussed by Berk and colleagues, DA transmission is certainly not the sole underlying factor for neural dysfunction in BD; other neurotransmitters are likely to play a role. Even though the current model is far from able to explain all pathology associated with BD, the current literature is not incompatible with a major role of DA. Therefore, the model used may be regarded as an interesting scientific starting point for epidemiological research on the extent and nature of any involvement of DA in the bipolar spectrum.

Fourth and final, the direct effects of lithium or antipsychotics dose adjustments on tardive movement disorders are complicated. Tardive dyskinesia has been reported to abate after dose increases [34], usually to reappear after some time, whereas acute dystonia often emerges following dose increases [5]. In order to avoid confounding, analyses were controlled for various relevant treatment-related variables, including changes in treatment doses. Even with this adjustment, one could question the extent to which dose adjustments may have influenced our results.

## Conclusion

Apart from the well-known association with antipsychotics, development of TDD was associated with various other dopamine proxy measures, indirectly supporting the notion of generalized dopamine dysregulation in BD.

## Competing interests

Inge van Rossum is employed by Lilly and has no further interests.

Diederik Tenback has received honoraria related to time and expertise devoted to Lilly and BMS advisory boards.

Jim van Os has received honoraria related to time and expertise devoted to EMBLEM study design and data analysis from Eli Lilly. He is/has also been an unrestricted research grant holder with, or received financial compensation as an independent symposium speaker from Eli Lilly, BMS, Lundbeck, Organon, Janssen-Cilag, GSK, AstraZeneca, Pfizer and Servier.

## Authors' contributions

IvR has been involved in the conception of the hypotheses, the statistical analyses, interpretation of the data and has drafted the manuscript. DT has been involved in the conception of the hypotheses, interpretation of the data and has reviewed the manuscript. JvO has been involved in the conception of the hypotheses, the statistical analyses, interpretation of the data and has reviewed the manuscript. All authors have given final approval of the version to be published.

## Pre-publication history

The pre-publication history for this paper can be accessed here:


